# Soybean 40S Ribosomal Protein S8 (GmRPS8) Interacts with 6K1 Protein and Contributes to Soybean Susceptibility to *Soybean Mosaic Virus*

**DOI:** 10.3390/v15122362

**Published:** 2023-11-30

**Authors:** Ting Hu, Dongquan Guo, Bowen Li, Liqun Wang, Hui Liu, Jinlong Yin, Tongtong Jin, Hexiang Luan, Lei Sun, Mengzhuo Liu, Haijian Zhi, Kai Li

**Affiliations:** 1 Key Laboratory of Biology and Genetics Improvement of Soybean, Ministry of Agriculture/Zhongshan Biological Breeding Laboratory (ZSBBL)/National Innovation Platform for Soybean Breeding and Industry-Education Integration/State Key Laboratory of Crop Genetics & Germplasm Enhancement and Utilization/College of Agriculture, Nanjing Agricultural University, Nanjing 210095, China; huting911025@163.com (T.H.); libowen717@163.com (B.L.); wanglq1124@njau.edu.cn (L.W.); qinghuan313718@163.com (H.L.); yinjinlong0000@126.com (J.Y.); 2018201084@njau.edu.cn (T.J.); sunlei@njau.edu.cn (L.S.); lmz@stu.njau.edu.cn (M.L.); 2 Jilin Academy of Agricultural Sciences, Changchun 130033, China; xzgdq@126.com; 3 Institute of Plant Genetic Engineering, College of Life Science, Qingdao Agricultural University, Qingdao 266109, China; luanhexiang87@163.com

**Keywords:** disease susceptibility, growth, protein interaction, virus-induced gene silencing

## Abstract

*Soybean mosaic virus* (SMV), a member of *Potyvirus*, is the most destructive and widespread viral disease in soybean production. Our earlier studies identified a soybean 40S ribosomal protein S8 (GmRPS8) using the 6K1 protein of SMV as the bait to screen a soybean cDNA library. The present study aims to identify the interactions between GmRPS8 and SMV and characterize the role of GmRPS8 in SMV infection in soybean. Expression analysis showed higher SMV-induced GmRPS8 expression levels in a susceptible soybean cultivar when compared with a resistant cultivar, suggesting that GmRPS8 was involved in the response to SMV in soybean. Subcellular localization showed that GmRPS8 was localized in the nucleus. Moreover, the yeast two-hybrid (Y2H) experiments showed that GmRPS8 only interacted with 6K1 among the eleven proteins encoded by SMV. The interaction between GmRPS8 and 6K1 was further verified by a bimolecular fluorescence complementation (BiFC) assay, and the interaction was localized in the nucleus. Furthermore, knockdown of GmRPS8 by a virus-induced gene silencing (VIGS) system retarded the growth and development of soybeans and inhibited the accumulation of SMV in soybeans. Together, these results showed that GmRPS8 interacts with 6K1 and contributes to soybean susceptibility to SMV. Our findings provide new insights for understanding the role of GmRPS8 in the SMV infection cycle, which could help reveal potyviral replication mechanisms.

## 1. Introduction

Soybean (*Glycine max* (L.) Merr.) is a key source of plant proteins and lipids and is an important commercial and cereal crop all over the world. However, *soybean mosaic virus* (SMV), a member of the *Potyvirus* family, is the most destructive and widespread viral disease in soybeans and leads to serious damage to soybean yield and seed quality [1,2,3]. Similar to other potyviruses, the genome of SMV is a single positive-sense RNA molecule of approximately 10 kb and encodes eleven proteins, namely protein 1 (P1), helper component-proteinase (HC-Pro), protein 3 (P3), P3N-PIPO, 6K1, cylindrical inclusion (CI), 6K2, viral protein genome-linked (VPg), nuclear inclusion a proteinase (NIa-Pro), nuclear inclusion b (NIb), and coat protein (CP) [4,5]. Most potyviral proteins are multifunctional in the infection cycle, such as viral translation and replication, assembly, encapsidation, cell-cell movement, long-distance movement, virulence and pathogenicity, and RNA silencing suppression [6,7,8,9,10,11,12,13,14,15,16,17,18,19]. Among the eleven proteins, 6K1 is recruited by 6K2 to the viral replication complex (VRC), is required for potyviral replication, and may play a role in viral movement [20,21,22,23].

Based on the different responses to a set of soybean cultivars, the SMV isolates were grouped into twenty-two SMV strains (SC1-SC22) in China and seven SMV strains (G1-G7) in the United States [24,25,26]. The most economical and effective approaches for managing losses from SMV are to plant virus-free seeds and breed cultivars containing single or multiple resistant genes [27]. Resistance genes can be categorized as dominant or recessive on the basis of their inheritance. Dominant resistance gene loci to SMV have been mapped to chromosomes 2, 13, and 14, respectively [28,29,30]. These dominant resistance genes can effectively inhibit viral infection, but they usually cannot resist all the SMV strains. Most recessive resistance genes are involved in viral infection and replication; the absence or mutation of recessive resistance genes can induce recessive resistance against plant viruses. Such translation initiation factors, particularly the eIF4E and eIF4G protein families, were confirmed to be the essential determinants in the RNA virus infection outcome and are the most widely used recessive resistance genes in several crop varieties [31,32]. Transgenic soybeans silenced for eIF4E showed broad resistance to three potyviruses, namely SMV, *Bean common mosaic virus* (BCMV), and *Watermelon mosaic virus* (WMV) [33]. Therefore, the exploration and utilization of recessive resistance genes is significant for breeding a resistant soybean cultivar.

Ribosomes are present in organisms ranging from bacteria to higher animals and are responsible for protein synthesis, which translates genetic information encoded by messenger RNA (mRNA) into proteins [34]. The eukaryotic 80S ribosomes are comprised of a small (40S) subunit and a large (60S) subunit. A number of ribosomal proteins (RPs) combine with ribosomal RNA (rRNA) to form the ribosomal subunits. RPs have been identified and named by their different protein molecular weights and their association with ribosomal subunits [35]. The 40S ribosomal subunit consists of 18S rRNA and 33 different RPs; it binds initiation factors to initiate protein synthesis [36]. RPs play essential roles in ribosome assembly and protein translation; mutations of RPs may retard cell growth and perturb the development of eukaryotes [37]. In addition, previous studies have shown the function of RPs in the infection of pathogens. Knockdown of RPS3 enhanced *classical swine fever virus* (CSFV) replication, and overexpression of RPS3 inhibited CSFV replication [38]. RPS18 is a maternal immune factor that can protect the early embryos of zebrafish from bacterial infection [39]. Five ribosomal protein genes (i.e., RPS2, RPS6, RPL7, RPL13, and RPL19) are required for the accumulation of *Turnip mosaic virus* (TuMV) [40,41]. However, limited information is available on the biological function and mechanism of most RPs in pathogen infection.

Approximately 40S ribosomal protein S8 (RPS8) is a structural protein of the ribosomal 40S subunit. RPS8 was first discovered in rat livers and is present in a messenger ribonucleoprotein granule complex containing untranslated mRNAs [42,43]. RPS8 was shown to be a rate-limiting factor in translational regulation and contributes to the cold-adaptability of rice [44,45]. However, the function of RPS8 in the virus infection of plants remains unclear. Using the SMV 6K1 protein as the bait to screen a soybean cDNA library by the yeast two-hybrid (Y2H) method, we identified a soybean RPS8 protein encoded by the gene Glyma.03g086400 [46]. In the present study, we conducted experiments to analyze the expression pattern of GmRPS8 induced by SMV, to examine its subcellular location in *Nicotiana benthamiana*, and to determine its protein-protein interactions with SMV. Furthermore, functional characterization of GmRPS8 by the virus-induced gene silencing (VIGS) system showed that the silencing of RPS8 decreased the accumulation of SMV in soybean. Our results demonstrated that GmRPS8 interacts with the 6K1 protein and contributes to soybean susceptibility to SMV, which could improve our knowledge of the mechanisms of potyviral infection in plants.

## 2. Materials and Methods

### 2.1. Bioinformatic Analyses

The RPS8 amino acid sequences of 20 plants were obtained from the NCBI database (https://www.ncbi.nlm.nih.gov/, accessed on 10 February 2023) and aligned by the DNAMAN 8.0 software. Protein physicochemical properties were predicted by the EXPASY server (https://web.expasy.org/protparam/, accessed on 15 October 2022). The protein domain was analyzed using the SMART website (https://smart.embl.de/, accessed on 15 October 2022). 

### 2.2. Plant Growth and Virus Strains

The soybean cultivars Nannong 1138-2 (SMV-SC3-susceptible) and Kefeng No.1 (SMV-SC3-resistant) were grown in a greenhouse at 25 °C day and 20 °C night with a photoperiod of 16 h. The *N. benthamiana* plants were grown in a greenhouse at 23 °C day and 20 °C night, with a photoperiod of 16 h. The relative humidity in the greenhouse is 60%, and artificial light was used to provide a light source (100 µM photons/s/m^2^). The SMV strain SC3 and the infectious clone pSC3-YFP were used in this study. pSC3-YFP was constructed from SMV strain SC3, which was confirmed to systemically infect Nannong 1138-2 [47]. All materials were stored at the Soybean Improvement Center of Nanjing Agricultural University (Nanjing, China).

### 2.3. Gene Cloning, Vector Construction and Primers

The total RNA was extracted from the soybean leaves infected by SMV strain SC3 using the TRIzol reagent (Vazyme, Nanjing, China) and was used to synthesize the first-strand cDNA using the Prime Script RT Reagent Kit (TaKaRa, Dalian, China). The coding sequences of GmRPS8 and the eleven genes of SMV were cloned from the cDNA using specific primers and were used to construct vectors.

To construct the vectors used for the Y2H assay, the coding sequences of GmRPS8 and the eleven genes of SMV were fused to pGBKT7 and pGADT7 by the homologous recombination method, respectively. To develop the vector used for subcellular localization, the coding sequence of GmRPS8 was merged into the pBin-GFP plasmid by the homologous recombination method. The vectors used in the BiFC assay were constructed using Gateway technology [48], and the interaction proteins were merged into the vectors pEarleyGate202-YN and pEarleyGate201-YC, respectively. To construct the VIGS vector for silencing GmRPS8, a 234-nt sequence region from GmRPS8 was inserted into the pBPMV-IA-V2 plasmid by the homologous recombination method to construct the recombinant vector pBPMV-IA-V2-RPS8. All the recombinant plasmids were verified by DNA sequencing. All primers used in this study are listed in Table S1.

### 2.4. RNA Extraction, Quantitative RT-PCR and ELISA

To analyze the expression of GmRPS8 induced by SMV, soybean seedlings of Nannong 1138-2 and Kefeng No.1 were inoculated with SC3 or phosphate buffered saline (PBS, 0.01 M, pH 7.4) at the vegetative stage of cotyledon. Before formal inoculation experiments, SC3 was multiplied by Nannong 1138-2. Young, symptomatic leaves were ground in PBS with a sterilized mortar and pestle. Fully expanded primary leaves of soybean plants Nannong 1138-2 and Kefeng No.1 were mechanically inoculated by gently rubbing with SC3 or PBS using a paintbrush. The leaves inoculated with PBS or SMV strain SC3 of Nannong 1138-2 and Kefeng No.1 were collected at 0, 6, 12, 24, 48, 72, 96, and 120 h post-inoculation (hpi). Each sample was independently collected and stored at −80 °C immediately after freezing in liquid nitrogen.

The total RNA of each sample was extracted using the TRIzol reagent (Vazyme, Nanjing, China) and reverse transcribed using the HiScript^®^ II QRT SuperMix (Vazyme Bio-Tech, Nanjing, China). qRT-PCR was performed using the 2 × SYBR Green Master Mix (Vazyme, Nanjing, China) on a CFX96 realtime PCR detection system (Bio-Rad, Hercules, CA, USA). The soybean gene *Tubulin* (accession No. AY907703) was used as the reference gene to normalize the cDNA. Transcript levels were quantified by the relative quantification (2^−ΔΔCt^) method [49]. Each sample was tested in three technical and biological replicates. ELISA was performed with an antibody diagnostic kit (V094-R2, Nanodiaincs, Fayetteville, NC, USA). The absorbance value of each sample at 405 nm was tested using Infinite 200PRO (TECAN, Männedorf, Switzerland).

### 2.5. Yeast Two-Yeast Assay

The Y2H assay was carried out according to the manufacturer’s protocols (Clontech, Palo Alto, CA, USA), and the yeast strain AH109 was used in this study. The bait vector pGBKT7-RPS8 and prey vectors were co-transformed into yeast cells and grown on SD/-Leu/-Trp and SD/-Leu/-Trp/-His/-Ade agar plates at 30 °C for 2–4 days.

### 2.6. Subcellular Localization and BiFC Assays

For the subcellular localization of GmRPS8, the recombinant plasmid pBin-RPS8-GFP was introduced into *Agrobacterium tumefaciens* strain EHA105 via electroporation. The agrobacterial culture contained relevant plasmids that were grown overnight on YEB mediums with kanamycin and rifampicin at 28 °C. The culture was pelleted by centrifugation and subsequently resuspended to an optical density of 0.8 at 600 nm (OD_600_) in infiltration buffer (10 mM MgCl_2_ [pH 5.6], 10 mM MES, and 150 μM acetosyringone), and then the cell suspensions were infiltrated into one-month-old *N. benthamiana* plants. H_2_B-mCherry was used as the nucleus marker. For the BiFC assay, RPS8 and 6K1 were fused into pEarleyGate202-YN and pEarleyGate201-YC, respectively, and individually transformed into *Agrobacterium tumefaciens* strain EHA105. Then, a mixture of two agrobacterial cultures containing relevant expression vectors was transfected into one-month-old *N. benthamiana* leaves. The interactions between 6K1-YC and YN and RPS8-YN and YC were used as the negative controls. Two days after agroinfiltration, a fluorescence signal was observed by confocal microscopy. The experiment was repeated at least three times.

### 2.7. Silencing of GmRPS8

The silencing of GmRPS8 using the *Bean pod mottle virus* (BPMV)-mediated VIGS system was performed as described before [50]. The constructs pBPMV-IA-V2-RPS8 and pBPMV-IA-R1M were mixed with a ratio of 1:1 and then mechanically inoculated on the primary leaves of Nannong 1138-2 treated for 24 h in the dark to obtain the RPS8-silenced (S_RPS8_) plants. Nannong 1138-2, inoculated with pBPMV-IA-V2 and pBPMV-IA-R1M, was used as the control (V) plant. The symptomatic leaves were used for RT-PCR to detect the silencing vectors. The leaves of V and S_RPS8_ plants were mechanically inoculated into the soybean cultivar Nannong 1138-2, and then pSC3-YFP was inoculated in the upper leaves with mosaic symptoms to analyze the function of GmRPS8. Twelve days post-inoculation, the YFP signal on the systemic leaves was observed under normal ultraviolet (UV) light. At the same time, systemic leaves were collected from the V and S_RPS8_ plants to detect the accumulation of SMV by qRT-PCR and ELISA assays.

### 2.8. Statistical Analysis

All measurements were biologically repeated three times. The soybean seeds of Nannong 1138-2 and Kefeng No.1 were planted in plastic pots (inside diameter 14 cm, height 12 cm, bottom diameter 10 cm), and a total of 15–20 seeds were planted in each pot and used for analysis. A Student’s *t*-test was used for significance analysis with SPSS Statistics 20 (SPSS Inc., Chicago, IL, USA).

## 3. Results

### 3.1. Characterization of the RPS8 Gene in Soybean

The GmRPS8 genomic sequence comprises four introns and five exons. The 663 bp coding sequence of GmRPS8 was cloned from the cDNA of Nannong1138-2 soybean plants and sequenced (Figure S1A), which matched the reference sequence in the Soybase database. The deduced protein consists of 220 amino acids with a molecular weight of 24.83 kDa and a theoretical isoelectric point (PI) of 10.37. GmRPS8 contains a Ribosomal_S8e domain at amino acids 1 and 200 (Figure S1B). Next, we investigated the conserved motifs among GmRPS8 and other RPS8 proteins in plants. Amino acid sequences of 20 RPS8 proteins from monocots (i.e., rice, maize, and wheat) and dicots (e.g., soybean, Arabidopsis, maize, tobacco, pepper, and tomato) were retrieved from the NCBI database, and their basic information is shown in Table S2. A multi-sequence alignment was conducted with these RPS8 proteins, and the results showed that GmRPS8 is highly similar to other RPS8 proteins, and the identities of these proteins are up to 90.09%, indicating that RPS8 proteins are highly conserved in plants (Figure S1C).

**Figure 1:**
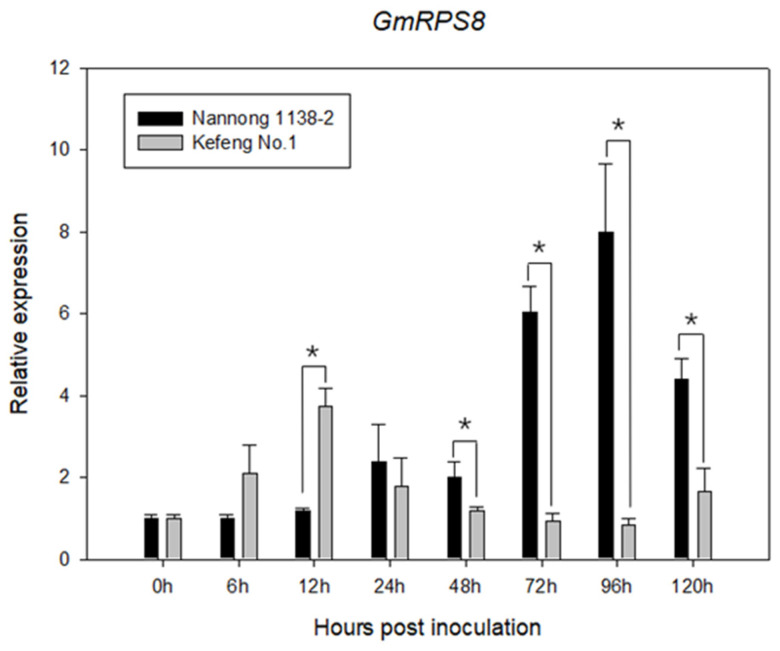
Expression analysis of GmRPS8 induced by SMV in soybean qRT-PCR was used to examine the expression levels of GmRPS8 in Nannong 1138-2 and Kefeng No.1 after SMV infection at 0, 6, 12, 24, 48, 72, 96, and 120 h post inoculation (hpi). Y-axe indicates the ratios of relative expression levels between samples inoculated with SMV and samples inoculated with phosphate buffered saline (PBS), and X-axe indicates the hours post-inoculation. The significant differences between susceptible and resistant soybean plants were tested by Student’s *t*-test, and the asterisk (*) represents *p* < 0.05. The experiment was repeated three times. Error bars indicate the standard deviation (SD).

To investigate whether GmRPS8 is involved in the response to SMV, the expression of GmRPS8 in soybean leaves infected by SMV strain SC3 was examined by qRT-PCR (Figure 1). After SMV infection, the expression levels of GmRPS8 were significantly increased in the susceptible Nannong 1138-2 plants, with a maximum expression of approximately eightfold at 96 h post-inoculation (hpi). In resistant Kefeng No.1 plants, GmRPS8 expression levels were upregulated from 6 to 24 hpi, with a maximum expression of approximately fourfold at 12 hpi, and then returned to their normal level after 48 hpi, suggesting that GmRPS8 was involved in the early stage of SMV infection in Kefeng No.1. Obviously, the expression of GmRPS8 induced by SMV in Nannong 1128-2 was higher than that in Kefeng No.1. These results indicated the involvement of GmRPS8 in the response to SMV in soybeans, especially in the susceptible soybean cultivar.

### 3.2. Subcellular Location of GmRPS8 and Analysis of Protein-Protein Interaction with SMV

**Figure 2:**
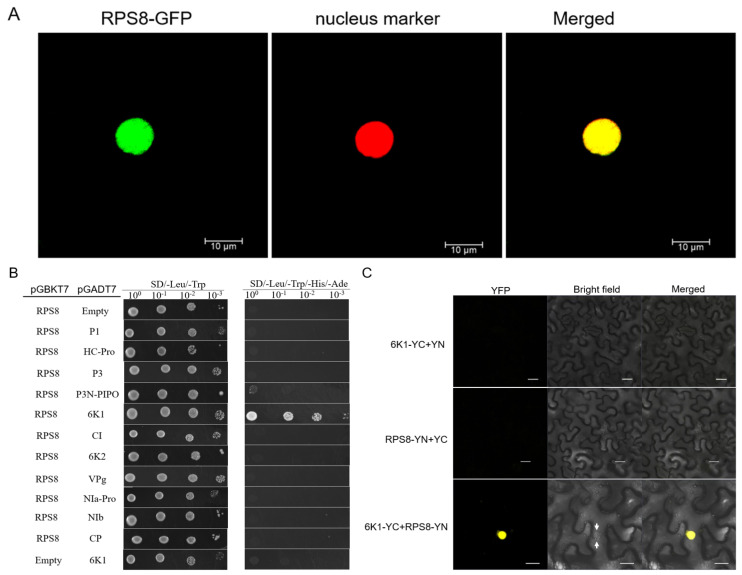
Subcellular location of GmRPS8 and analysis of protein-protein interaction with SMV (**A**) Subcellular localization of GmRPS8 in *Nicotiana benthamiana* epidermal cells (pBin−RPS8−GFP) was co-agroinfiltrated with a nucleus marker into the leaves of one-month-old *N. benthamiana* plants. Scale bars are 10 µm. (**B**) Analyses of the interactions between GmRPS8 and the eleven proteins of SMV in the yeast two-hybrid (Y2H) system Yeast cells co-transformed with pGBKT7-RPS8 + pGADT7 and pGBKT7 + pGADT7-6K1 were used as the negative controls. The yeast-grown cells were diluted 1-, 10-, 100-, and 1000-fold. The Y2H experiment was repeated three times. (**C**) Verifying the interaction between RPS8 and 6K1 by bimolecular fluorescence complementation (BiFC) assay, RPS8-YN and 6K1-YC were co-agroinfiltrated into the leaves of one-month-old *N. benthamiana* plants. The interaction between 6K1-YC and YN and RPS8-YN and YC was used as the negative control. The structure shown by the arrows was probably a nucleus. Scale bars are 20 μm.

To examine the subcellular location of GmRPS8 in planta, a fusion vector pBin-RPS8-GFP was constructed and transiently expressed in *N. benthamiana* epidermal cells. Two days after agroinfiltration, the agroinfiltrated leaves were observed by confocal microscopy. The results showed that the RPS8-GFP fusion protein was located in the nucleus (Figure 2A).

The interactions between GmRPS8 and the eleven proteins encoded by SMV (i.e., P1, HC-Pro, P3, P3N-PIPO, 6K1, CI, 6K2, VPg, NIa-Pro, NIb, and CP) were analyzed by Y2H assay. The coding sequence of the GmRPS8 gene was amplified from Nannong 1138-2 and merged into the vector pGBKT7 (with the DNA-binding domain of GAL4 (BD)) to generate the bait vector pGBKT7-RPS8. The eleven viral genes were amplified from SMV strain SC3 and separately fused into the vector pGADT7 (with the DNA activation domain of GAL4 (AD)) to generate the prey vectors. As shown in Figure 2B, yeast cells co-transformed with pGBKT7-RPS8 and pGADT7-6K1 were able to grow on the SD/-Leu/-Trp/-His/-Ade agar plates, while other combinations could not, indicating that RPS8 only interacts with the 6K1 protein among the eleven proteins of SMV. Then, the interaction between RPS8 and 6K1 was further confirmed by a bimolecular fluorescence complementation (BiFC) assay (Figure 2C). The results showed that RPS8 interacted with 6K1 in leaf cells of *N. benthamiana,* and the structure shown by arrows suggested that the interaction was probably in the nucleus. These data indicated that GmRPS8 interacts with the 6K1 protein.

### 3.3. Silencing of GmRPS8 Retarded the Growth and Development of Soybean

**Figure 3:**
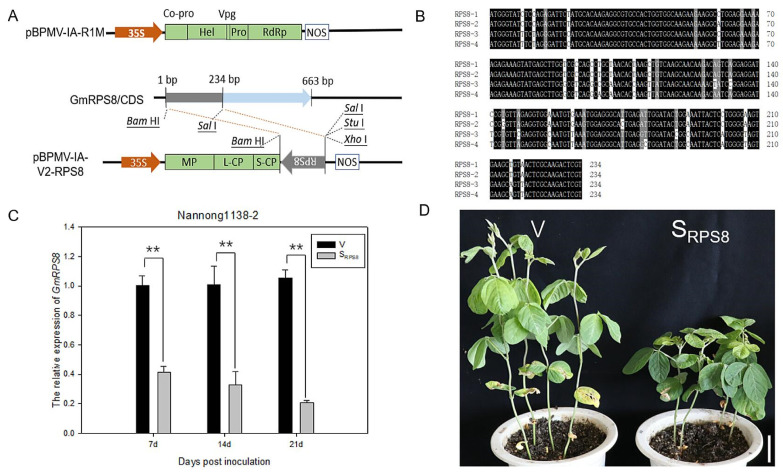
Silencing GmRPS8 retarded the growth and development of soybeans. (**A**) Schematic drawing of the *Bean pod mottle virus* (BPMV)-mediated virus-induced gene silencing (VIGS) system A 234-bp fragment of GmRPS8 was inserted in antisense orientation behind the S-CP gene. RNA1 and RNA2 of the BPMV genome are driven and terminated by the Cauliflower mosaic virus 35S promoter and NOS terminator, respectively. (**B**) A sequence alignment of the 234-bp fragments of four GmRPS8 genes: RPS8-1 (Glyma.03G086400), RPS8-2 (Glyma.16G087700), RPS8-3 (Glyma.18G157300), and RPS8-4 (Glyma.08G346500). Sequence alignment was carried out using the BioXM 2.7 program; identical residues are shaded in black, and conserved residues are marked in gray. (**C**) The silencing efficiency of GmRPS8 in the RPS8-silenced (S_RPS8_) plants was analyzed by qRT-PCR at 7, 14, and 21 days post-inoculation (dpi). The significant differences between V and S_RPS8_ plants were tested by Student’s *t*-test, and double asterisks (**) represent *p* < 0.01. The experiment was repeated three times. Error bars indicate SD. (**D**) Morphological phenotypes of the V and S_RPS8_ plants at 15 dpi. Scale bars are 5 cm.

The VIGS assay was adopted using a BPMV-mediated vector system to investigate the function of GmRPS8. A 234-bp fragment of the GmRPS8 gene was fused with the pBPMV-IA-V2 plasmid to generate the VIGS construct pBPMV-IA-V2-RPS8 (Figure 3A). Based on the sequence homology and the analysis of the SGN VIGS Tool (https://vigs.solgenomics.net/, accessed on 20 March 2022), the construct pBPMV-IA-V2-RPS8 probably targets two genes simultaneously (Glyma.03G086400 and Glyma.16G087700) and potentially even targets four soybean RPS8 genes (Figure 3B and Table S3), and a primer pair qRT-S_RPS8_-F/R that measures combined expression levels of the four genes was designed to determine the silencing efficiency of GmRPS8. Then, the empty vector pBPMV-IA-V2 and the recombinant vector pBPMV-IA-V2-RPS8 were individually co-inoculated with pBPMV-IA-R1M on the leaves of the soybean cultivar Nannong 1138-2 to obtain the control (V) and RPS8-silenced (S_RPS8_) plants. The silencing efficiency of GmRPS8 in Nannong1138-2 was analyzed by qRT-PCR. Compared with the V plants, the expression levels of GmRPS8 were obviously reduced by approximately 59%, 67%, and 79% at 7, 14, and 21 days post inoculation (dpi) in S_RPS8_ plants, respectively, indicating that the silencing of GmRPS8 was effective (Figure 3C). Morphological phenotypes of the V and S_RPS8_ plants were observed at 15 dpi. Compared with the V plants, the silencing of GmRPS8 seriously affected the growth and development of soybean plants, resulting in plant dwarfism and growth retardation (Figure 3D).

### 3.4. Silencing of GmRPS8 Limited the Accumulation of SMV in Soybean

**Figure 4:**
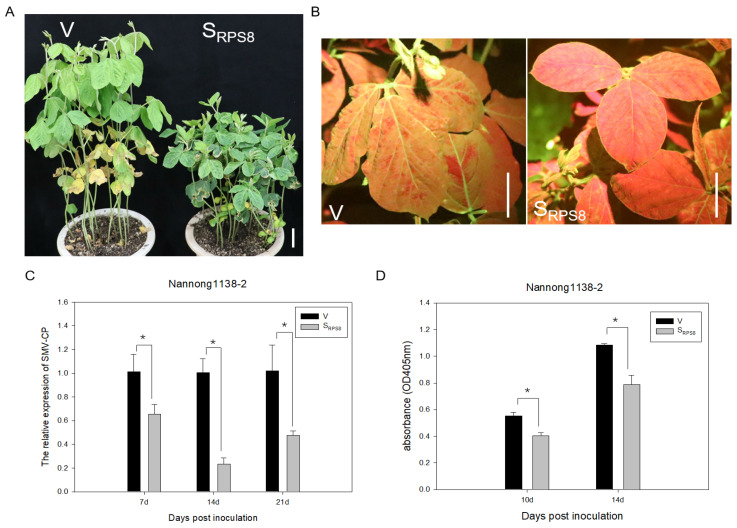
Silencing GmRPS8 limited the accumulation of SMV in soybeans. (**A**) Morphological phenotypes of the V and S_RPS8_ plants of Nannong 1138-2 at 12 days post-inoculation with pSC3-YFP. Scale bars are 5 cm. (**B**) Leaves of the V and S_RPS8_ plants inoculated with pSC3-YFP were observed under normal UV light at 12 dpi. Scale bars are 5 cm. (**C**) Analysis of the relative expression levels of SMV-CP in the V and S_RPS8_ plants by qRT-PCR at 7, 14, and 21 dpi (**D**) The accumulation of SMV in the V and S_RPS8_ plants was detected at 10 and 14 dpi by ELISA. The experiment was repeated three times. Error bars indicate SD. The significant differences between V and S_RPS8_ plants were tested by Student’s *t*-test, and the asterisk (*) represents *p* < 0.05.

Next, we investigated whether the silencing of GmRPS8 affected soybean resistance to SMV. The infectious clone pSC3-YFP (virulent on Nannong 1138-2) constructed from SMV strain SC3 was inoculated into the V and S_RPS8_ plants of Nannong 1138-2. Twelve days after inoculation (dpi), observation of the SMV-inoculated plants revealed that the silencing of GmRPS8 resulted in leaf curling, plant dwarfing, and growth retardation compared to the V plants (Figure 4A). At the same time, we observed the YFP fluorescence signal in the systemic leaves under normal UV light, which reflected the content of SMV. Compared with the V plants, there was obviously less YFP signal in the S_RPS8_ plants (Figure 4B). Furthermore, the accumulation of SMV on systemic leaves was detected by qRT-PCR and ELISA assays. The qRT-PCR results showed that the SMV accumulation in S_RPS8_ plants was reduced by approximately 35%, 77%, and 53% at 7, 14, and 21 dpi, respectively, compared with that in the V plants (Figure 4C). The results of qRT-PCR were further confirmed by the ELISA results, which showed less SMV content in the S_RPS8_ plants compared to the V plants (Figure 4D). Together, the silencing of GmRPS8 reduced the accumulation of SMV in soybeans, indicating that GmRPS8 contributes to soybean susceptibility to SMV.

## 4. Discussion

Our earlier studies have identified a soybean RPS8 protein using 6K1 as the bait to screen a soybean cDNA library, suggesting the potential role of GmRPS8 in modulating SMV infection [46]. Currently, limited information is available on the functional relationship between RPS8 and virus infection. In this study, we characterized the RPS8 protein of soybean. The general structure of ribosomes and the mechanisms of ribosome biogenesis in eukaryotes are evolutionarily conserved, and the RPs of cytosolic ribosomes in eukaryotes are highly conserved in general [51,52]. Our results showed that the RPS8 proteins from dicots and monocots are highly conserved in plants (Figure S1C), implying similar functions for RPS8 in plants. After SMV infection, SMV virions would robustly replicate and accumulate in soybean susceptible cultivars, while a small amount of SMV could be detected in the inoculated leaves of soybean resistant cultivars at the early stage of infection. Although the resistant cultivar showed extreme resistance to viruses, a slower host response allows a certain degree of virus replication and movement before conferring virus arrest [53,54]. After SMV infection, the expression of GmRPS8 was induced at the early stage of infection in the resistant Kefeng No.1 plants, while significantly upregulated in the susceptible Nannong 1138-2 plants (Figure 1), indicating that the expression of GmRPS8 was related to SMV replication.

SMV-6K1 localizes to the nucleus and cytoplasm; the interaction between 6K1 and RPS8 was verified to be negative using the Y2H method based on the DUALmembrane system [46]. Subcellular localization showed that GmRPS8 was located in the nucleus (Figure 2A), which is not exactly consistent with the localization of human RPS8 in the nucleus and cytoplasm in Hela cells [44]. Therefore, the identification of interactions by the Y2H method based on the GAL4 nuclear system may be necessary, since the interactions occurring in the nucleus may not be detected in the DUALmembrane system. Here, we examined the interactions between GmRPS8 and the eleven proteins of SMV by the Y2H assay based on the GAL4 nuclear system, and results showed that GmRPS8 only interacted with 6K1 among the eleven viral proteins (Figure 2B). Moreover, the interaction between 6K1 and GmRPS8 was further confirmed by the BiFC assay, and the interaction probably occurred in the nucleus (Figure 2C), which strongly supports our hypothesis that the interactions occurring in the nucleus may not be detected by the Y2H method of the DUALmembrane system.

Ribosome biogenesis and protein translation are closely associated with cell growth, differentiation, and the development of organisms. Ribosomal proteins are essential for the assembly and function of ribosomal subunits; mutations in ribosomal proteins can severely retard the cell growth of plants, animals, or microorganisms [37]. Mutant mice lacking RPL29 displayed global skeletal growth defects and lower postnatal viability [55]. Silencing of the RPS6 gene family resulted in serious effects on plant development, such as chlorotic leaves, upward leaf curling, and plant growth retardation in *N. benthamiana* [41]. RPS10 promotes shoot branching mainly by promoting the development of axillary shoot, and the mutation of RPS10 affects meristematic function in *A. thaliana* [56]. Silencing of PsRPs26 suppressed hyphae formation and fungal colonization, indicating the involvement of PsRPs26 in fungi development during infection [57]. In this study, we identified the function of GmRPS8 in soybeans by the BPMV-mediated VIGS system and discovered that GmRPS8 was efficiently knocked down by about 60–80% in soybean plants (Figure 3C).The soybean plants with GmRPS8 knocked down showed plant dwarfing and growth retardation (Figure 3D), indicating that RPS8 is essential in the growth and development of plants. Moreover, RPs are involved in a variety of pathological processes. The 40S ribosomal subunit contains 33 different RPs, namely RPS0-RPS31 and RACK1 [34,36]. A few 40S ribosomal proteins were reported to positively or negatively regulate the resistance to pathogens, including RPS2, RPS3, RPS6, RPS18, and RACK1 [38,39,40,41,58]. Previous studies have shown that RPS8 is a rate-limiting factor in translational regulation and contributes to the cold-adaptability of rice [44,45]. However, the function of RPS8 in virus infection in plants has not been reported. Here, we showed that the silencing of GmRPS8 significantly reduced the accumulation of SMV in soybean (Figure 4), suggesting that RPS8 is a positive regulator of potyvirus infection. The general defects in translation machinery and the corresponding effects on plant growth and development may lead to the inhibition of virus accumulation, but this is less likely. The TRV VIGS system was used to silence RPS6, RPL19, RPL13, RPL7, and RPS2, respectively, resulting in severe developmental defects in *N. benthamiana* to varying degrees, but the severity of the silencing phenotype did not necessarily correspond to the inhibition of virus accumulation. For example, the RPL19-silenced plants had a less severe phenotype than the RPS6-silenced plants, but the RPL19-silenced plants were almost immune to TuMV infection [40].

Plant viruses do not contain functional ribosomes and other components required for translation in their virions, and their genomes do not encode components for the translational machinery, so they have evolved a variety of strategies to recruit the host translation devices to quickly and efficiently translate the viral genome [59]. Numerous host factors involved in potyviral infection have been identified, such as five ribosomal proteins (i.e., RPS2, RPS6, RPL7, RPL13, and RPL19), the eIF4F complex (eIF4E, eIF4G, and eIF4A), poly(A) binding proteins (PABPs), eukaryotic translation elongation factor 1A (eEF1A), and three 60S ribosomal subunits: P1, P2, and P3, and Ribosomal Protein P0 [40,60,61,62,63,64]. Previous studies have shown that potyviral 6K1 protein is recruited by 6K2 to the viral replication complex and is required for potyviral replication [21,22]. However, the precise function and mechanism of 6K1 in viral replication remain unclear. Here, we confirmed that among the eleven proteins encoded by SMV, GmRPS8 only interacted with 6K1 in the nucleus, and the silencing of GmRPS8 significantly reduced the accumulation of SMV in soybean. Thus, we speculated that RPS8 was a host factor recruited by the potyviral 6K1 protein and promoted viral replication in plants, which could help to reveal the potyviral replication mechanisms.

## 5. Conclusions

In this study, GmRPS8 was identified and functionally characterized. GmRPS8 is located in the nucleus, and GmRPS8 only interacts with 6K1 among the eleven proteins encoded by the SMV genome. Furthermore, the knockdown of GmRPS8 seriously affected the growth of soybeans and significantly reduced the accumulation of SMV in soybeans, suggesting that GmRPS8 is a positive regulator of SMV infection.In conclusion, we indicated that GmRPS8 interacts with the 6K1 protein and contributes to soybean susceptibility to SMV. Future experiments that focus on the complex mechanisms of how GmRPS8 promotes viral replication are expected.

## Data Availability

All data are provided in the manuscript and its Supplementary Materials.

## Supplementary Materials

The following supporting information can be downloaded at: https://www.mdpi.com/article/10.3390/v15122362/s1, Figure S1: Characterization of the RPS8 gene in soybean; Table S1: Primers used in this study; Table S2: Basic information of the 20 RPS8 proteins in plants; and Table S3: Target genes of pBPMV-IA-V2-RPS8 analyzed by the SGN VIGS Tool.
