# Supplementary material for: Soybean 40S Ribosomal Protein S8 (GmRPS8) Interacts with 6K1 Protein and Contributes to Soybean Susceptibility to Soybean Mosaic Virus
Source: Viruses. 2023 Nov 30;15(12):2362. doi: 10.3390/v15122362 (PMC10748009; doi:10.3390/v15122362)
Supplement: Supplementary file 1 [file viruses-15-02362-s001.zip › viruses-2717432-supplementary.pdf]

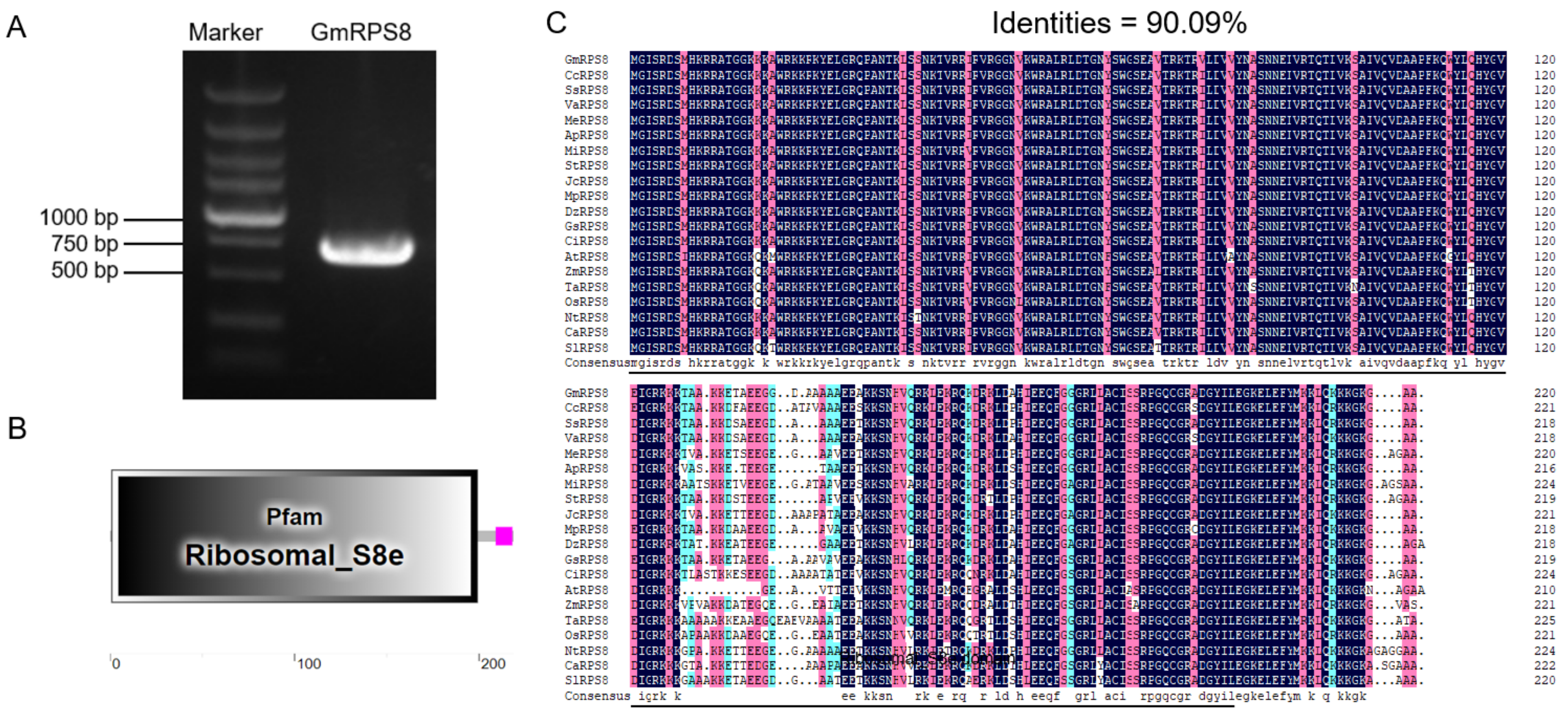

Figure S1. Characterization of the RPS8 gene in soybean. (**A**) Gene cloning of GmRPS8. Marker: 5000 DNA marker. (**B**) Schematic structures of GmRPS8. The rectangle represents the Ribosomal_S8e domain and the pink bar depicts the low complexity. (**C**) A multi-sequence alignment of RPS8 protein sequences of 20 plants (i.e. *Glycine max*, *Cajanus cajan* (L.) Millsp, *Vigna angularis* (Willd.) Ohwi et Ohashi, *Spatholobus suberectus* Dunn, *Manihot esculenta* Crantz, *Abrus precatorius* L., *Mangifera indica* L., *Senna tora* (L.) Roxb., *Jatropha curcas* L., *Mucuna pruriens* (L.) DC., *Durio zibethinus* Murr., *Glycine soja* Siebold & Zucc., *Carya illinoinensi* (Wangenheim) K. Koch, *Arabidopsis thaliana* (L.) Heynh., *Zea mays* L., *Triticum aestivum* L., *Oryza sativa* L., *Nicotiana tabacum* L., *Capsicum annuum* L., *Solanum lycopersicum* L.). The Ribosomal_S8e domain is underlined. The identical amino acid residues among the 20 RPS8 proteins are shaded in dark blue. The amino acid residues marked in pink and light blue represent identities of 75% and 50% among the 20 RPS8 proteins , respectively.

Table S1. Primers used in the study.

Primer name	Sequences (5′→3′)
pBin-RPS8-F	ATTTCAGAAGAAGATCTGGGTACCATGGGTATCTCCAGAGATTCTATGC
pBin-RPS8-R	CTCGCCCTTGCTCACCATGGATCCAGCAGCACCCTTTCCCTTCTT
Gateway-RPS8-F	GGGGACAAGTTTGTACAAAAAAGCAGGCTTCATGGGTATCTCCAGAGATTCTATGC
Gateway-RPS8-R	GGGGACCACTTTGTACAAGAAAGCTGGGTCAGCAGCACCCTTTCCCTTCTT
Gateway-6K1-F	GGGGACAAGTTTGTACAAAAAAGCAGGCTTCATGGCCAAAACAGCAACTCAATT
Gateway-6K1-R	GGGGACCACTTTGTACAAGAAAGCTGGGTCCTGCACTTTAACATCCTCAC
AD-6K1-F	GTACCAGATTACGCTCATATGATGGCCAAAACAGCAACTCAATT
AD-6K1-R	CAGCTCGAGCTCGATGGATCCCTGCACTTTAACATCCTCAC
AD-6K2-F	GTACCAGATTACGCTCATATGATGAGCAAACATGAGGTGAGC
AD-6K2-R	CAGCTCGAGCTCGATGGATCCTTGAGTTGATACTGGCTC
AD-P1-F	GTACCAGATTACGCTCATATGATGGCAACAATCATGATTG
AD-P1-R	CAGCTCGAGCTCGATGGATCCGTAATGCTGAACATCCTC
AD-Hc-Pro-F	GTACCAGATTACGCTCATATGATGTCTCAAACTCCTGAAGCCCAG
AD-Hc-Pro-R	CAGCTCGAGCTCGATGGATCCACCAACTCTATAAAATTTCATCTCACTC
AD-P3-F	GTACCAGATTACGCTCATATGATGGGGGAAGTGCAACAAAGG
AD-P3-R	CAGCTCGAGCTCGATGGATCCCTGTGCGGAGACATCTTCTG
AD-P3N-PIPO-F	GTACCAGATTACGCTCATATGATGGGGGAAGTGCAACAAAGG
AD-P3N-PIPO-R	CAGCTCGAGCTCGATGGATCCCATTTTTGGAAAAGTGTATTT
AD-CI-F	GTACCAGATTACGCTCATATGATGAGTCTTGATGAGATTCAAAACATAG
AD-CI-R	CAGCTCGAGCTCGATGGATCCCTGTAGTTGAACTGCATTTAAAATGC
AD-VPg-F	GTACCAGATTACGCTCATATGATGGGAAAGAAGAGGCAGATAC
AD-VPg-R	CAGCTCGAGCTCGATGGATCCTTCCATTTCAACCCTTTC
AD-NIa-Pro-F	GTACCAGATTACGCTCATATGATGAGCAAATCTGTCTACAAAG
AD-NIa-Pro-R	CAGCTCGAGCTCGATGGATCCTTGTACTGTCACTGTAC
AD-NIb-F	GTACCAGATTACGCTCATATGATGGGGAAGAAGGAAAGATG
AD-NIb-R	CAGCTCGAGCTCGATGGATCCTTGTAAGGAAACTGATTC
AD-CP-F	GTACCAGATTACGCTCATATGATGTCAGGCAAGGAGAAAGAAGGAG
AD-CP-R	CAGCTCGAGCTCGATGGATCCCTGCTGTGGGCCCATGC

Primer name	Sequences (5′→3′)
BD-RPS8-F	CATATGGCCATGGAGGCCGAATTCATGGGTATCTCCAGAGATTCTATGC
BD-RPS8-R	CGGCCGCTGCAGGTCGACGGATCCAGCAGCACCCTTTCCCTTCTT
qRT-Tubulin-F	GGAGTTCACAGAGGCAGAG
qRT-Tubulin-R	CACTTACGCATCACATAGCA
qRT-SMV-CP-F	TTCTGAAAGTCCATATATGCCTAG
qRT-SMV-CP-R	GCCTTTCAGTATTTTCGGAGTT
qRT-RPS8-F	AGCTTGTGCGTACTCAGACT
qRT-RPS8-R	CACCACCAAACTGCTCCTCA
qRT-S_{RPS8}-F	GAGAAGCGCCAGAAAGATCG
qRT-S_{RPS8}-R	AGCAGCACCCTTTCCCTTCTTCTT
pBPMV-IA-V2-F	TGACATTCTCCTGGGAATTTCCC
pBPMV-IA-V2-R	CACACTTCACACATCATTACGAC
V2-RPS8-F	GAATCCTCTGCATGAGGATCCACGAGTCTTGCGAGTTACAGCTTCA
V2-RPS8-R	CTCTCGAGGCCTGGAGTCGACATGGGTATCTCCAGAGATTCTATGC

Table S2. Basic information of the 20 RPS8 proteins in plants.

Protein name	Accession No.	Species	Description	Identity (%)	PI
GmRPS8	XP_003520991.1	*Glycine max*	40S ribosomal protein S8	100	10.37
CcRPS8	XP_020238484.1	*Cajanus cajan*	40S ribosomal protein S8	95	10.4
VaRPS8	XP_017440765.1	*Vigna angularis*	40S ribosomal protein S8-like	91	10.37
SsRPS8	TKY68689.1	*Spatholobus suberectus*	40S ribosomal protein S8	90	10.4
MeRPS8	XP_021633425.1	*Manihot esculenta*	40S ribosomal protein S8	89	10.4
ApRPS8	XP_027335163.1	*Abrus precatorius*	40S ribosomal protein S8-like	90	10.4
MiRPS8	XP_044502964.1	*Mangifera indica*	40S ribosomal protein S8-like	90	10.4
StRPS8	KAF7845602.1	*Senna tora*	40S ribosomal protein S8-like	88	10.39
JcRPS8	XP_012085188.1	*Jatropha curcas*	40S ribosomal protein S8	94	10.37
MpRPS8	RDX64930.1	*Mucuna pruriens*	40S ribosomal protein S8	90	10.3
DzRPS8	XP_022759807.1	*Durio zibethinus*	40S ribosomal protein S8-like	89	10.44
GsRPS8	XP_028207931.1	*Glycine soja*	40S ribosomal protein S8-like	93	10.42
CiRPS8	XP_042949348.1	*Carya illinoinensis*	40S ribosomal protein S8-like	90	10.44
AtRPS8	NP_200732.2	*Arabidopsis thaliana*	Ribosomal protein S8e family protein	82	10.49
ZmRPS8	NP_001132384.1	*Zea mays*	40S ribosomal protein S8-like	88	10.35
TaRPS8	XP_044332410.1	*Triticum aestivum*	40S ribosomal protein S8-like	84	10.45
OsRPS8	XP_015636562.1	*Oryza sativa*	40S ribosomal protein S8-like	89	10.41
NtRPS8	XP_016485119.1	*Nicotiana tabacum*	40S ribosomal protein S8-like	93	10.35
CaRPS8	XP_016575949.2	*Capsicum annuum*	40S ribosomal protein S8	93	10.33
SlRPS8	XP_004242427.1	*Solanum lycopersicum*	40S ribosomal protein S8	85	10.3

Table S3. Target genes of pBPMV-IA-V2-RPS8 analyzed by SGN VIGS Tool.

Gene	Matches	Functional Description
Glyma.03G086400.1	213	Ribosomal protein S8e family protein
Glyma.16G087700.1	131	Ribosomal protein S8e family protein
Glyma.18G157300.1	24	Ribosomal protein S8e family protein
Glyma.08G346500.1	12	Ribosomal protein S8e family protein
